# Elucidating the Structure, Dynamics, and Interaction of a Choline Chloride and Citric Acid Based Eutectic System by Spectroscopic and Molecular Modeling Investigations

**DOI:** 10.1021/acsomega.3c04570

**Published:** 2023-10-04

**Authors:** Md Ackas Ali, Md Abdul Kaium, Sayed Nesar Uddin, Md Jaish Uddin, Oluseyi Olawuyi, Albert D. Campbell, Carl Jacky Saint-Louis, Mohammad A. Halim

**Affiliations:** † Department of Chemistry and Biochemistry, Kennesaw State University, Kennesaw, Georgia 30144, United States; ‡ Division of Quantum Chemistry, The Red-Green Research Center, BICCB, 16, Tejkunipara, Tejgaon, Dhaka 1215, Bangladesh

## Abstract

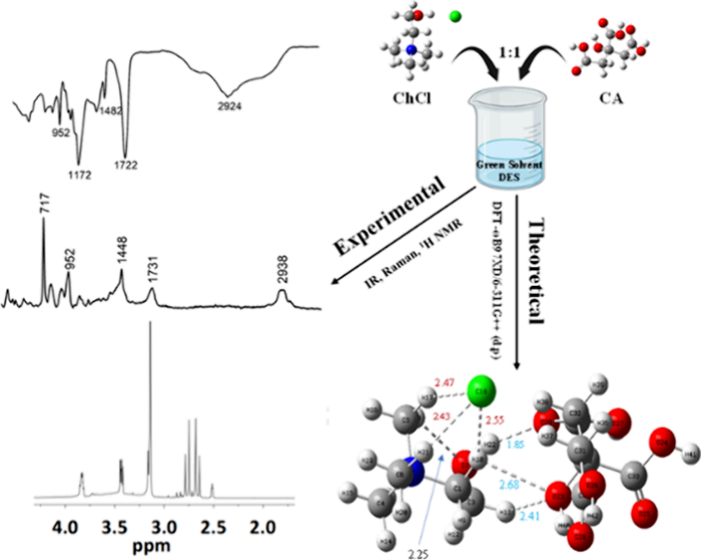

Eutectic solvent systems are versatile solvents that have found widespread use in numerous applications. Traditional solvents are homogeneous, having only one component, and their chemistry is relatively simple, with some exceptions. On the other hand, deep eutectic solvents (DESs) comprise binary components, generally a donor and an acceptor in hydrogen bonding with varying ratios. The interaction chemistry among the donor and acceptor involved in hydrogen bonding in DESs is complicated. Although numerous research is focused on the synthesis and application of DESs, few studies are reported to elucidate the complex structure and dynamic and interaction behavior of DESs. In this study, we employed calorimetry, vibrational spectroscopy techniques including FTIR and Raman, and nuclear magnetic resonance to derive insight into the structural feature and noncovalent contact of choline chloride (ChCl) and citric acid (CA) while they formed DESs. The 1:1 ChCl/CA eutectic system showed phase transitions and melting peaks with the most pronounced peak at 156.22 °C, suggesting the DESs melting at a lower temperature than the melting temperatures of ChCl and CA. In addition to IR and Raman findings, ^1^H NMR investigations demonstrate hydrogen bonding intermolecular interactions between ChCl and CA, supporting the formation of 1:1 ChCl/CA DESs based on the deshielded chemical shifts of the proton for Ch. The interaction of the chloride anion with the methyl protons (H4) and methylene protons (H3) of ChCl as well as the strong hydrogen bonding interactions between the hydroxyl hydrogen (H1) of ChCl with one of CA’s carbonyl oxygens both supported the formation of conformer E. In addition, molecular dynamics followed by the density functional theory (DFT) was employed to visualize the structure and interaction of DESs using the ωB97XD theory and 6-311++G (d,p) basis set. Both experimental and theoretical IR, Raman, and structural analyses provided evidence of the formation of DESs by possessing hydrogen bonds. These multifaceted experimental and computational investigations provide details of structural and intermolecular interactions of ChCl/CA DESs.

## Introduction

Eutectic solvent systems typically contain binary components, in contrast to the sole component found in traditional solvents. In eutectic solvents, one component should be a hydrogen bonding acceptor termed as HBA, and another component must be a hydrogen bonding acceptor denoted as HBD. Intermolecular hydrogen bonds as well as other noncovalent bonding between the HBA and HBD are assumed to play important roles on the formation of DESs, resulting in a decrease in melting point to room temperature.^1,2^ The high thermal stabilities, moderate volatility and vapor pressure, tunable polarity, and nonflammability are reasons why DESs are considered a novel classification of ionic liquids (ILs).^3,4^ Despite their similarity, DESs and ILs are recognized as two different solvents.

Various classes of DESs have been successfully synthesized thus far.^2,5,6^ Choline chloride (ChCl)-based DESs, conversely, have demonstrated to be particularly effective because of their low cost, biodegradability, and minimal toxicity.^7,8^ Electrochemistry, biochemistry, materials science, and petrochemical engineering are just a few of the many fields that have found widespread use for these DESs as solvents or reaction media.^9−11^ Although there has been substantial progress in the study of DESs, the combined experimental and theoretical investigations carried out to obtain the structure and interaction of DESs are limited. These features are widely recognized as being crucial to future DES research and application.^12^ As DESs have multiple components, the noncovalent bonding between these components is more intricate than those seen in simpler solvents. In the molecular structures and the accompanying physical properties, theoretical approaches can be an invaluable resource.^13,14^

Various analytical techniques have been used to characterize and investigate the structure and dynamics of DESs. The composition, functional group shifting, water content, impurities, and transport phenomena of DESs are investigated by NMR.^15,16^ However, the structural characterization of DESs by NMR has been less explored. The characterizations of DESs to confirm the intermolecular interactions, hydrogen bond formation, water quantification, and temperature effect and to detect the low-frequency vibration modes are largely performed by FTIR and Raman spectroscopy.^17−19^ The study of vibrational analysis is crucial for assigning vibration modes, which can aid in the interpretation of experimental data and related phenomena.^20^ Souza et al., for example, conducted an experimental FTIR investigation of ChCl/urea DESs. The vibrational modes and mechanism of the different DESs where ChCl was used as hydrogen bonding acceptor together with various hydrogen bond donors such as ethylene glycol, malonic acid, and glycerol were studied by Perkins et al. using molecular dynamics (MD) simulations.^21^ Particularly interesting is the comparison between the FTIR spectrum obtained by Zhang et al. of ChCl/magnesium chloride hexahydrate and the calculated IR spectrum computed by the employed B3LYP theory and 6-311++G(d) basis set.^22^ In comprehending the properties of DESs, Shafie et al. examined various molar ratios of citric acid (CA) monohydrate and ChCl, including 3:1, 2:1, 1:1, 1:2, and 1:3. The deep eutectic points of DESs with a 1:1 molar ratio were observed, and they exhibited the lowest temperature of melting.^23,24^ Furthermore, FTIR analysis revealed that the functional groups of each component of the DESs were shifted because of hydrogen bonding.^25^ Additionally, the application of the calorimetry technique is of significance in the context of a eutectic solvent system, as it enables the assessment of their thermal characteristics, encompassing phase transitions and melting points. Abbott et al. reported the formation of a eutectic mixture through the combination of ChCl and carboxylic acids. They utilized multiple techniques, including scanning calorimetry technique, to analyze the thermal characteristics of various DES systems.^26^

Most of the studies on DESs have been based on experiments to find the deep eutectic point in a certain molar ratio, and there has been limited research on their structural insight and intermolecular interactions with DESs, specifically ChCl/CA DES.^24^ The absence of a molecular-level understanding is a crucial problem because it impedes the optimization of DESs for various applications. The applications of these solvents are associated with the structure, interactions between the components, and properties. That is why it is important to have a fundamental grasp of the interactions between these components. In this study, various experimental techniques and computational simulations were employed to reveal the structural insight of the ChCl/CA DES system.

## Experimental Details

### Materials

In this study, the chemicals were used as provided without further purification, and the quality of all analytical reagents was taken. The specific chemicals used were choline chloride (ChCl, 99%, Acros Organics) and citric acid (CA, 99.6%, ACS reagent, anhydrous, Acros Organics).

### Preparation of DESs

To synthesize DESs, five different ratios (1:1, 1:2, 1:3, 2:1, and 3:1 ChCl/CA) of the hydrogen bonding acceptor (choline chloride) and hydrogen bonding donor (citric acid) were combined in a covered beaker. The reaction was carried out in an oil bath maintained at 80 °C while stirring the mixture at a rate of 700 rpm. To perform a precise investigation of the DESs, DSC, ATR-IR, and Raman spectroscopy experiments were conducted on the fresh samples. This was crucial because of the very hygroscopic nature of the components.

### Differential Scanning Calorimetry (DSC)

In this study, a DSC60 apparatus from SHIMADZU Corporation, Japan, operated in the Heat Flux system, was used for the experiments.^27^ In the presence of an argon atmosphere and employing a liquid-nitrogen cooling system, standard calibration procedures were conducted with indium at a flow rate of 35 mL min^–1^. The DES sample (1:1 ChCl/CA) was added in pans made of aluminum and sealed in an airtight manner at a dosage of approximately 10 mg per sample. The samples were equilibrated at 40 °C for 5 min and subsequently heated from 40 up to 17 °C, followed by an isothermal period of 5 min at 40 °C, and consequently cooled from 17 to 40 °C and thereafter cooled to 25 °C. The rate used for this process was set at 5 °C/min.

### Experimental ATR-FTIR and Raman

The DES spectra of all ratios were collected by using an attenuated total reflectance on a PerkinElmer 100 FTIR spectrophotometer. The IR spectra were acquired at the range from 400 to 4000 cm^–1^ of the ChCl, CA, and the investigated eutectic system at room temperature. The Raman scattering spectra were acquired with the use of a Raman spectrophotometer (a Mono Vista CRS+ for Spectroscopy and Imaging GmbH, Germany), which employed a 785 nm laser. All the same spectral data were further processed by using Origin Pro 2020 for adjustment, deconvolution, smoothing, and leveling of the baseline (Origin Lab, 2020).

### Experimental ^1^H Nuclear Magnetic Resonance (NMR)

The Bruker AM-400 spectrometers were employed to obtain the proton NMR spectra. The chemical shifts were conveyed in parts per million (δ scale) and were located downfield compared to tetramethylsilane (TMS). The remaining protium within the NMR solvents (δ 2.50 ppm with DMSO-*d*_6_) was used as a reference point. The preparation of the ^1^H NMR samples involved dissolving approximately 15 mg of the solids in 0.50 mL of DMSO-*d*_6_.

### Molecular Dynamics Simulation and Conformer Isolation

The YASARA dynamics suite was utilized to conduct the molecular dynamics (MD) simulation^28^ to produce the most possible cluster conformers of DESs for 1:1 ChCl/CA by considering the Gaff2 force field parameters that were autoutilized by YASARA.^29^ Gaff2 force field parameters for these molecules were also generated using the Autosmiles package followed by MD simulation for the comparison between these two methods. The results obtained from Figures S1 and S2 indicate that no substantial distinction is found between the methods employed with respect to total energy, Vdw energy, Coulombic energy, and interactions involved in DES system formation. So autoutilized force field parameters were considered for MD simulations. An 8.0 Å cutoff radius was utilized for the study of close-range van der Waals and Coulombic contacts. The particle-mesh Ewald (PME) approach was implemented to obtain extended-range electrostatic contacts. The simulation was conducted using the N (constant number), V (constant volume), and T (temperature) ensemble, with a periodic boundary box of 58.81 × 58.81 × 58.81 Å at 298 K temperature, where the steepest descent minimization technique was considered for the initial energy minimization.^30^ The short-range forces were revised at every 1.25 fs, whereas for the 2 fs of long-range forces was considered. The 50, 100, and 150 pairs of the ChCl and CA were considered to perform 50 ns simulation in the gas phase where the trajectories were recorded for analysis in 100 ps intervals. For the comparison among the pairs, the radial distribution functions of atom to atom in each of the three systems were analyzed. As the number of pairs varied, the peak distance was consistent but with varying probabilities (Figure S3). A system with lower complexity was utilized, and a subset of 50 pairs was chosen as a benchmark system for subsequent calculations. By observing every simulation snapshot of the above-mentioned ratio, 14 conformers were isolated based on the observed dominant interactions in the DES system. From the above-mentioned simulations in the gas phase, six of the cluster conformers of ChCl and CA were selected as the most robust based on SPE (single point energy) calculations. This selection was made from a total of 14 conformers listed in Table S1 using the HF (Hartree–Fock) theory by employing the 6-311++G (d,p) basis set. Figure 4 is a schematic illustration of the clusters that have been found to be the most favorable from an energetic standpoint. Although computational studies can provide detailed structural insights and noncovalent interactions of DESs, they also face some challenges including (i) which force fields or functionals or basis sets will be suitable for DESs systems, (ii) heavy computational costs for larger systems, (iii) selecting the initial geometry of the system, (iv) simulation parameters for liquid and gas, and (v) choosing the best sampling methods. These problems can be very complicated when experimental data are not available for DESs. A comprehensive study including various force fields, hybrids, and dispersion-corrected DFT functionals and better electron-correlated Gaussian-*n* theories requires a separate study that is beyond the current scope of this work.

### Quantum Calculations and Calculated IR and Raman

The density functional theory was employed to execute quantum calculations on a set of selected cluster conformers using the Gaussian09 software package.^31^ The calculations were carried out using the ωB97XD function with the basis set consisting of 6-311++G (d,p). The ωB97XD is a variant of Becke’s 97 functional that incorporates range separation and enables the inclusion of supplementary dispersion corrections. Moreover, the ωB97XD hybrid function is constituted by 22% HF substitution over the close-range region and 100% HF throughout the extended range. The utilization of the range-separated hybrid function is crucial in the examination of orbital energy estimation of a system with conjugated components that involves charge exchange.^32^ The study also investigated the thermodynamic properties such as Gibbs free energy and enthalpy for 1:1 ChCl/CA DES. The study utilized the ωB97XD/6-311++G (d,p) level of theory to conduct gas phase IR and Raman calculations for all cluster conformers. A vibrational scaling factor of 0.957 was utilized. Figure 5 displays the obtained spectra.

## Results and Discussion

### Differential Scanning Calorimetry (DSC)

**Figure 1:**
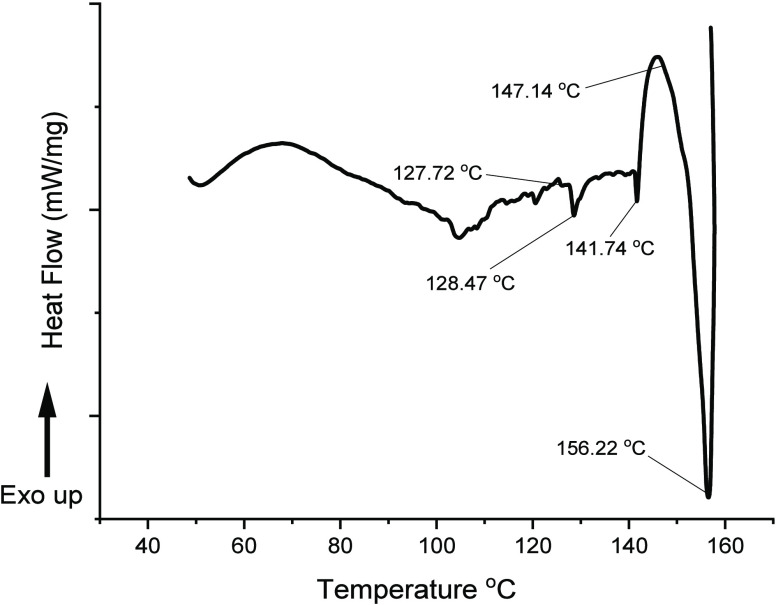
DSC curvature for 1:1 ChCl/CA deep eutectic solvents (DESs).

DSC analysis was carried out with 1:1 ChCl/CA eutectic system. Analyzing DSC data for the temperature range studied revealed phase transitions within the samples (Figure 1). A series of endothermic peaks, which signify melting, notably at around 128.47, 141.74, and 156.22 °C, were seen in the thermogram of the DES sample. The most intense of all of the noticeable endothermic peaks in the thermogram of ChCl/CA was the peak at 156.22 °C, the onset of which was seen at around 147.14 °C. This temperature range may indicate the point at which the DESs completely melted. A similar melting point was reported for 1:1 ChCl/CA by Abbott et al.^26^ As expected of a DES, this melting point was decreased compared to those of both the ChCl (302 °C) and citric acid (153 °C) that constituted the DES system. The close comparison of ChCl–organic acid–alcohol-based DESs, for example, 1:1 ChCl/MA (malic acid), with ChCl/CA has shown the melting point (K) at 131 °C. The interaction properties of 1:1 ChCl/MA and other ChCl-based deep eutectic solvents are similar to the 1:1 ChCl/CA eutectic system that we studied in our previous study.^33^

### Experimental ATR-FTIR and Raman

**Figure 2:**
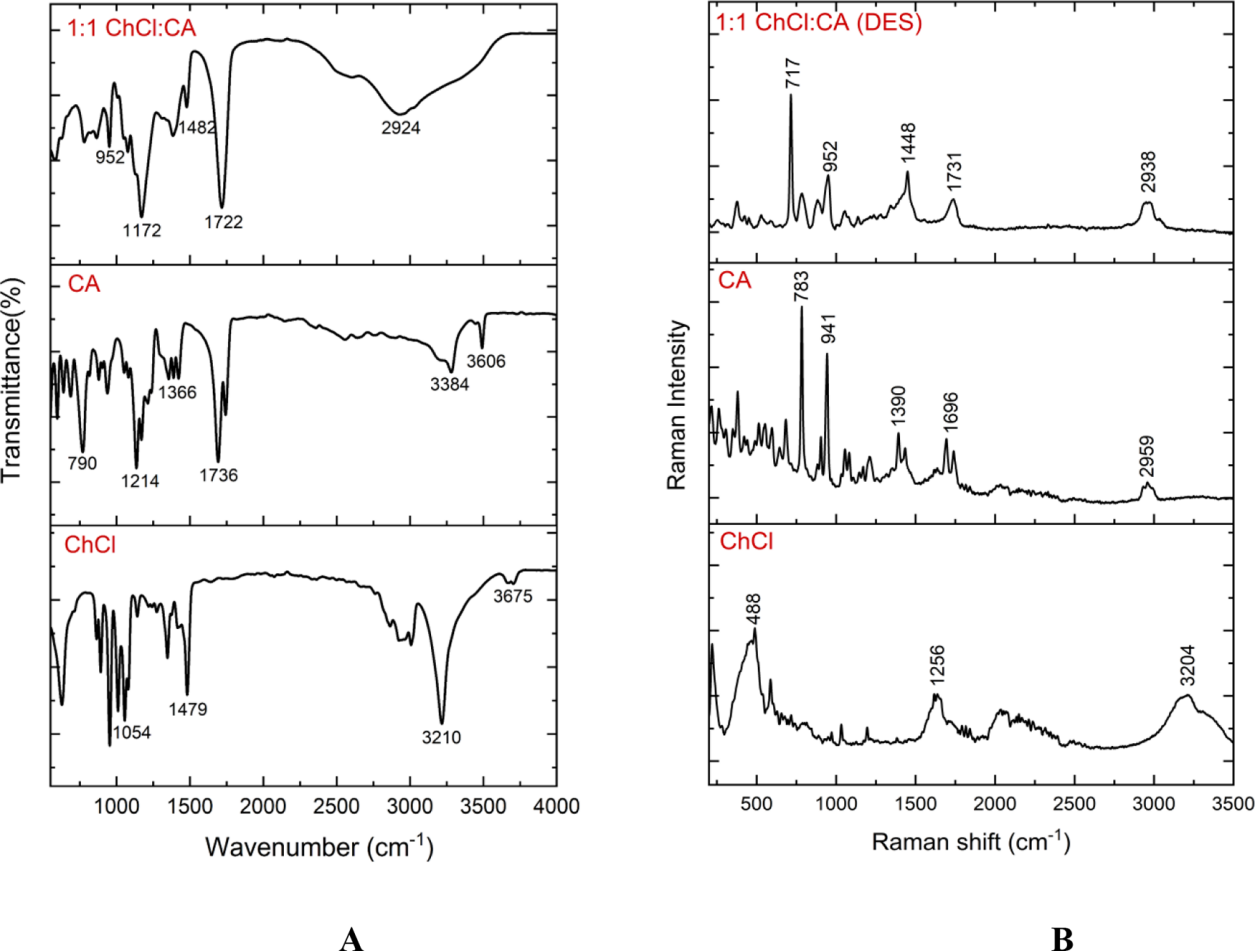
Experimental (A) ATR-FTIR and (B) Raman spectra of choline chloride (ChCl in the bottom), citric acid (CA in middle), and 1:1 ChCl: CA (DESs in top).

The study employed ATR-FTIR to examine the intermolecular interactions among the constituents of eutectic solvent systems (all five ratios) and their corresponding structural characteristics (Figure S4).^34^Figure 2A,B shows the spectra of ATR-FTIR and Raman for ChCl, CA, and the DESs of 1:1 ChCl/CA, respectively. The comparison of the IR peak shifts for the components in the DESs spectra is shown in Table 1. This identifies the component that makes the most contribution to the creation of hydrogen bonds. The peaks observed at 3675, 3606, and 2924 cm^–1^ on ChCl, CA, and DESs, respectively, are associated with the stretching of the O–H bond vibration. The formation of cluster conformers by H-bonding is demonstrated by the broad spectrum of O–H for DESs, which is determined by the shift of the peak at 2924 cm^–1^. The presence of characteristic −O–H peaks was found at 3675 and 3506 cm^–1^ for ChCl and CA, respectively. Hence, the DES cluster showed a wider −O–H peak at 2924 cm^–1^, which can be assigned to the hydrogen bonding interactions facilitated by ChCl and CA within the DES cluster. The C–H stretching vibrations were observed at 3210, 3384, and 2622 cm^–1^ for ChCl, CA, and DESs, respectively. The peak shifting for C–H stretching vibration for the DESs cluster is due to the H-bond formation between ChCl and CA. For pure CA, a peak associated with C=O stretching was detected at 1736 cm^–1^ where there is no peak for ChCl. The carbonyl (C=O) peak for the DESs changes to 1722 cm^–1^, which is also confirmed by the theoretical IR spectrum. It can be deduced from the greater proportion of peak shifting, indicating that the participation of CA is more substantial to the formation of conventional intermolecular hydrogen bonding. The all-representative IR frequencies are listed in Table 1.

**Table 1:** FTIR Representative Wavenumber and Band Assignment (ND = Not Detected)

components	wavenumber (cm^–1^)					
	stretching of O–H bond	stretching of CH_2_ bond	stretching of C=O bond	bending of CH_2_ bond	bending of CH_3_	stretching of C–O bond
ChCl	3675	3210	ND	1479	1054	ND
CA	3606	3384	1736	1366	ND	1214
eutectic system	2924	2622	1722	1482	952	1172

The 1:1 ChCl/CA DESs exhibited a significant new peak for C–H bending (−CH_3_) at 952 cm^–1^ in the fingerprint region, which was unique to the ChCl spectra and absent from the CA spectrum. This indicates the strong influence of ChCl on the cluster conformers. Furthermore, it can be observed that the CA spectrum exhibits a distinctive C–O stretching peak at 1214 cm^–1^. This peak is also apparent in the DESs spectrum, although with greater intensity and a lower spectral displacement. Overall, the spectral profile of the DESs displays a greater degree of similarity to that of the CA, with ChCl presenting a greater proportion of peak shift in the −OH_CA_ region, indicating the significant role played by CA in the development of DESs. This phenomenon represents the strong interaction network between ChCl and CA to form DESs and reveals the structural features of DESs. Shafie et al. also investigated various ratios of ChCl and CA DESs and reported the FTIR.^24^ In both FTIR investigations, 1:1 ChC/CA DES analysis exhibited notable shifts in characteristic peaks, such as the enlarged −O–H peak around ∼2924 cm^–1^, indicating strong hydrogen bonding interactions between ChCl and CA within the DES cluster. In addition, 1:1 ChCl/CA DESs exhibited distinctive spectral characteristics, such as the appearance of a distinct C–H bending peak at ∼952 cm^–1^ due to ChCl and a prominent C–O stretching peak at 1214 cm^–1^ attributed to CA, demonstrating the complex interaction and structural characteristics of ChCl and CA within the eutectic solvent.

The Raman spectra associated with stretching vibrations of −O–H are detected at 3204, 2959, and 2938 cm^–1^ for ChCl, CA, and DESs, respectively, and all representative Raman shifts are shown in Figure 2B. Similarly, one of the dominant vibrational modes for C=O stretching was identified for the same CA and DES components at 1696 and 1731 cm^–1^, respectively. Meanwhile, other prominent peaks of C–H at 1390 cm^–1^ are also witnessed in the CA spectrum, which shifted as a sharp intense peak in DES formation at 1448 cm^–1^. All these vibrational shifts in the DES cluster signify the compact construction of 1:1 ChCl/CA DES synthesis by engaging the strong H-bonding contact with strong participation from both ChCl and CA.

### Nuclear Magnetic Resonance (NMR)

**Figure 3:**
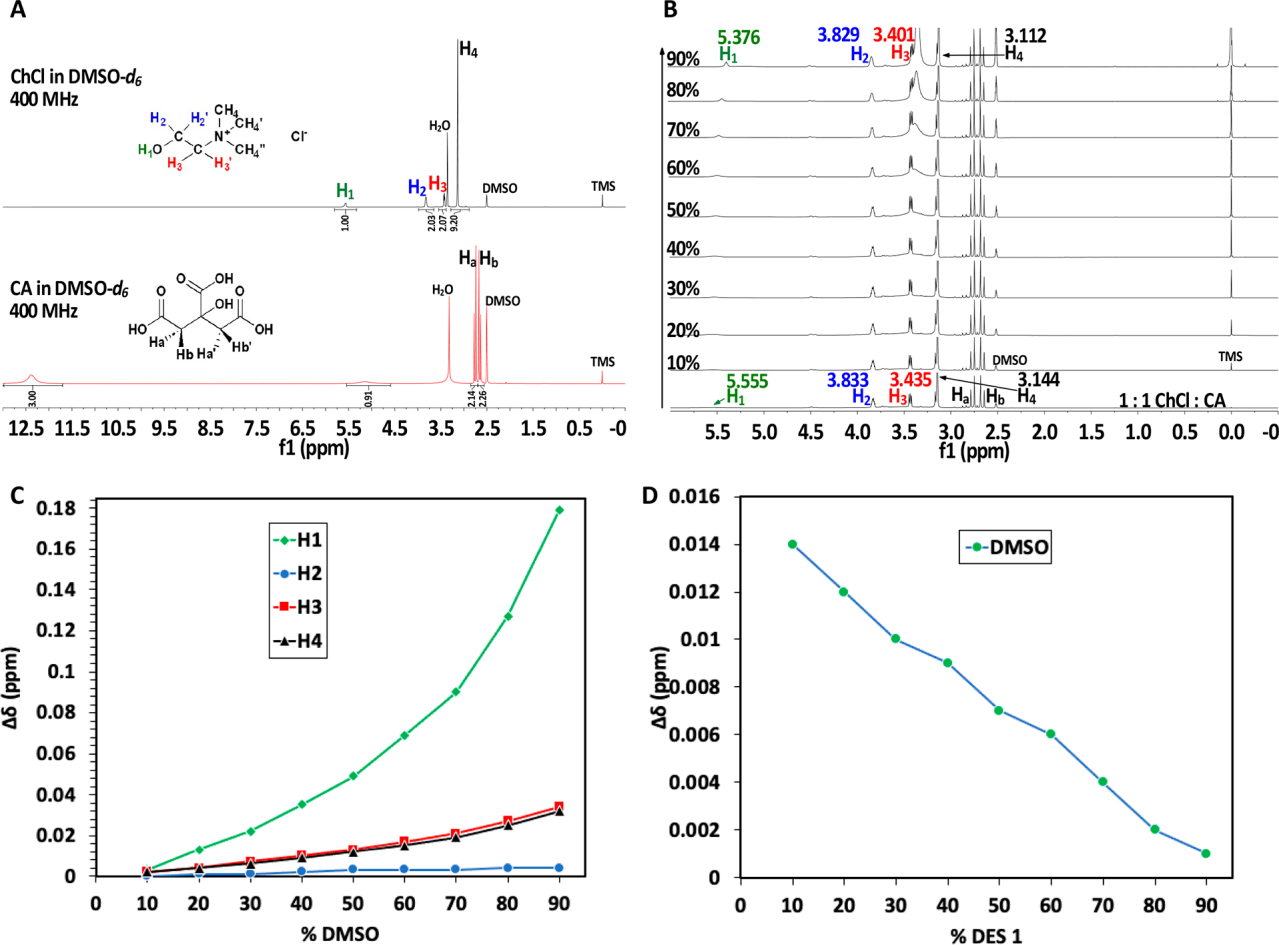
(A) ^1^H NMR assignments of pure ChCl and CA in DMSO-*d*_*6*_. (B) Dilution ^1^H NMR experiment of 1:1 ChCl/CA (DESs) with DMSO-*d*_*6*_. (C) Chemical shift variation (Δδ) of ChCl’s H_1_, H_2_, H_3_, and H_4_. (D) Chemical shift variation (Δδ) of methyl hydrogens in DMSO.

A dilution ^1^H NMR experiment of 1:1 ChCl/CA (DESs) with DMSO was performed to gain a better understanding of the intermolecular interactions between ChCl and CA. The ^1^H NMR assignments of pure ChCl and CA in DMSO-*d*_6_ are shown in Figure 3A, and their proton chemical shifts are consistent with published values.^35,36^ TMS (tetramethylsilane) was assigned a chemical shift of zero (0.00 ppm) and was used as the internal reference substance during the dilution ^1^H NMR experiment of DESs. To begin the experiment, a ^1^H NMR spectrum of DESs was obtained in DMSO-*d*_6_ (Figure 3B). The chemical shift of ChCl’s hydrogen atoms (H_1_, H_2_, H_3_, and H_4_) moves upfield as the DMSO concentration increases (from bottom to top) (Figure 3B,C). The difference in chemical shifts between stock solutions of DESs and those after 90% DMSO dilution is denoted as the chemical shift variation (Δδ). Although we found positive Δδ values for ChCl’s H_1_ (0.179 ppm), H_2_ (0.004 ppm), H_3_ (0.034 ppm), and H_4_ (0.032 ppm) (Figure 3C), we also found negative Δδ values for the hydrogen atoms of DMSO’s methyl groups (Figure 3D). This demonstrates that when DMSO-*d*_6_ is introduced into DESs, the hydrogen atoms of ChCl shifted upfield. This result also indicates that when DMSO-*d*_6_ is introduced to DESs, ChCl and CA migrate apart from one another, reducing their hydrogen bonding interactions. It is well established that when hydrogen atoms are drawn by electron-withdrawing elements or groups to form hydrogen bonds, they exhibit positive chemical shifts and travel downfield.^37,38^ This also implies that the greater the distance between a hydrogen atom and an electronegative element or group is, the more negative the chemical shift it will display and migrate upfield. This impact is readily seen in ChCl’s H_1_, H_2_, H_3_ and H_4_ as the DES sample is diluted with DMSO-*d*_6_. As the concentration of DMSO-*d*_6_ increases, both H_1_ and H_2_ shift upfield as a result of decreased hydrogen bonding to CA. This finding explains the interactions of H1 with one of CA’s carbonyl oxygens and H_2_ with CA’s hydroxyl oxygen observed in cluster E. Both H_3_ and H_4_ shifted upfield, resulting in reduced contact with the electronegative chloride anion (Cl^–^) and weaker hydrogen bond interactions. According to these findings, adding DMSO to DESs clearly influences the hydrogen bonding interactions between ChCl and CA. These findings support the development of cluster E and suggest that the chloride anion (Cl^–^) interacts with the methyl group of choline (HBD) and the −CH_2_ group of choline (Ch^+^).

### Structural Geometry and Hydrogen Bonding Analysis

**Figure 4:**
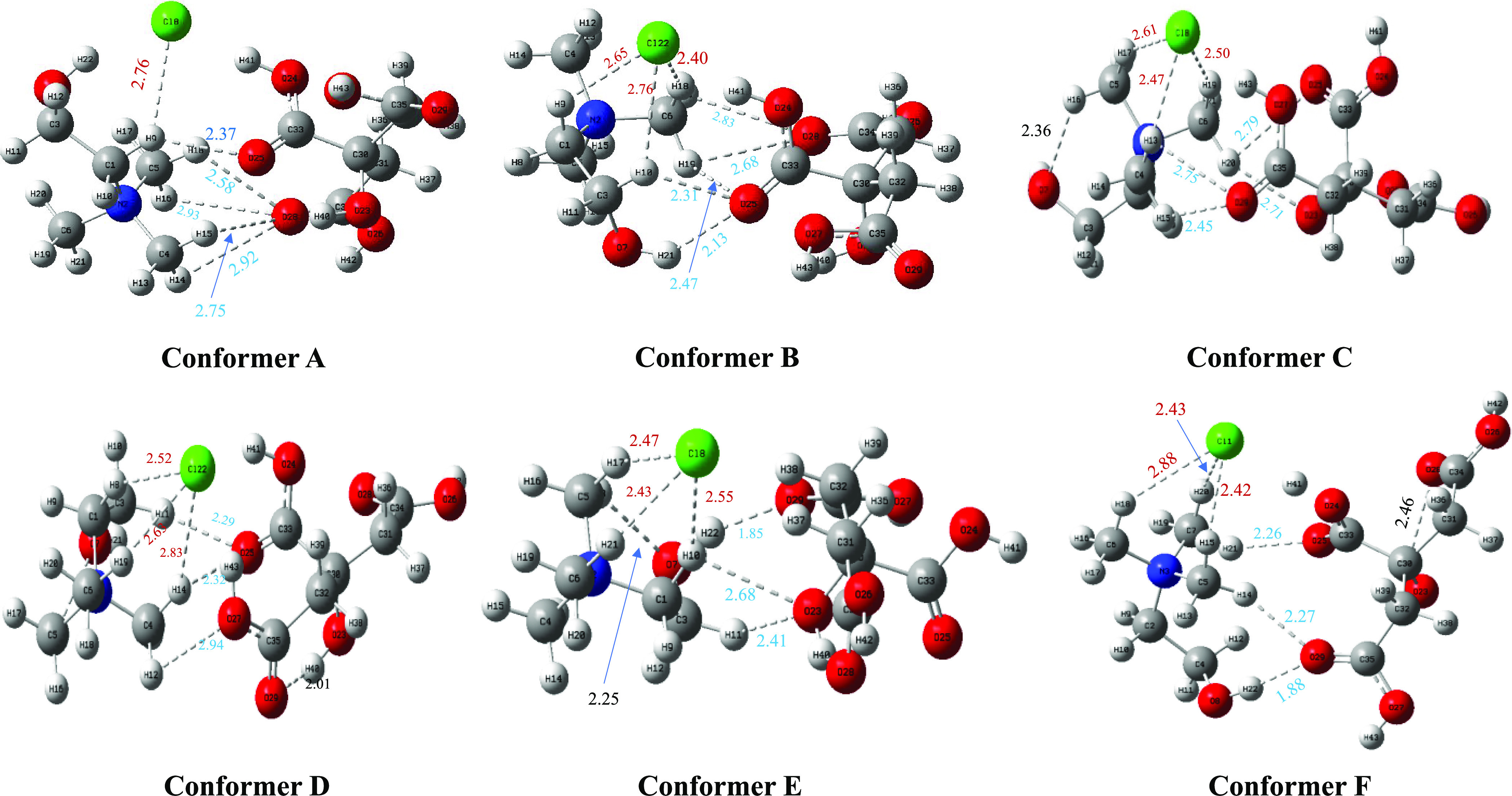
Structure of six selected conformers (A, B, C, D, E, and F) retrieved from MD simulation and subsequently optimized by the ωB97XD theory and a basis set of the 6-311++G(d,p) level of theory. In this context, the hydrogen atoms are represented by the color white, the carbon atoms are depicted in ash, the oxygen atoms are denoted by the color red, and the nitrogen atoms are indicated by the color blue. The red color denotes interaction distances between Cl····ChCl, the blue color represents the interaction distance between ChCl····CA, and the black color denotes H-bond distances Ch····Ch and CA····CA. Displayed distances are in Å units.

Initial conformers of 1:1 ChCl and CA have been generated from a 50 ns MD simulated structure of 50:50 ChCl/CA ratio for quantum calculation. After estimating the single point energy (SPE) of all 14 conformers (Table S1), we considered the six most stable candidates among them for further quantum calculations employing the ωB97XD theory and a basis set of 6-311++G (d,p). A pictorial illustration was given along with hydrogen bond distances of six cluster conformers labeled in Figure 4. The proper orientation of individual components within the DESs system is a crucial factor in comprehending the eutectic properties of these substances. The manner in which molecules interact with one another plays a vital role in outlining the physical characteristics of DESs, including but not limited to thermal stability, melting point, solubility, viscosity, conductivity, and vibrational spectra.^24,39,40^ In this DES, the chloride anion (Cl^–^) interacts with the choline (HBD) methyl group and −CH_2_ group of choline (Ch) and H-Bond distances ranging from 2.41 to 2.83 Å. In all DES cluster conformers, we observed an H-bond between one of the oxygens of the CA carboxylic group with C–H of Ch having a range from 2.17 to 2.93 Å. We also noticed a rare O–H_Ch_···O=C_CA_ conventional H-bond formed between Ch and CA in conformer at 1.1, 3.1, and 5.1 with distances of 2.13, 1.85, and 1.88 Å, respectively. This illustrates all possible orientations of the 1:1 ChCl/CA DES structure and provides insight into all of the possible interactions with the entire scenario. In our previous study, we studied different types of ChCl-based DES properties and interactions that are selected based on different hydrogen bond donor (HBD) molecules.^33^ We found that different ChCl complexes have distinct hydrogen bonding patterns involving N–H_HBD_···Cl and O–H_HBD_···Cl interactions, and the study also revealed the importance of intermolecular interactions between the −OH group of Ch^+^ and acceptor groups in the donor molecule. Hence, the 1:1 ChCl/CA DESs found that the Ch^+^ part of the ChCl is involved in more interactions and forms strong H-bonds with the CA, whereas the Cl^–^ is not evolving and forming interactions toward CA.

The data presented in Table 2 indicate that the conformers within the DES cluster demonstrate spontaneous enthalpy and Gibbs free energy changes. Furthermore, the hydrogen bonds present in these conformers demonstrate a robustness that allows for the stretching of the covalent bonds of ChCl and CA. Furthermore, the computed values of Δ*G*, Δ*E*, Δ*H*, and Δ*S* offer a valuable chemical understanding of the process of forming hydrogen bonds and the spontaneity of the computed cluster. In the gas phase, we measure a value of Δ*G* of −60.15 kJ/mol for conformer E, which has the highest degree of similarity to its individual components. Thus, according to Δ*G* values, conformer E is the cluster structure that most closely resembles its individual components, making it the most spontaneous. The cluster structures are formed spontaneously over a wide range of temperature, as shown by the Δ*S* values.

**Table 2:** Δ*G*, Δ*E*, and Δ*H* (kj/mol) of Choline Chloride (ChCl), Citric Acid (CA), and the DES Conformers Were Computed Utilizing the ωB97xD Theory and a Basis Set of 6-311++G(d,p)

parameter	ChCl	CA	eutectic-conformer A	eutectic-conformer B	eutectic-conformer C	eutectic-conformer D	eutectic-conformer E	eutectic-conformer
Δ*G* (kJ/mol)			–57.67	–56.82	–53.72	–58.17	–60.15	–54.07
Δ*E* (kJ/mol)			–7.22	–8.24	–6.81	–7.86	–10.49	–7.24
Δ*H* (kJ/mol)			–4.74	–5.76	–4.33	–5.38	–8.01	–4.76

### Calculated IR and Raman Spectra of Six DES (1:1 ChCl/CA) Conformers

**Figure 5:**
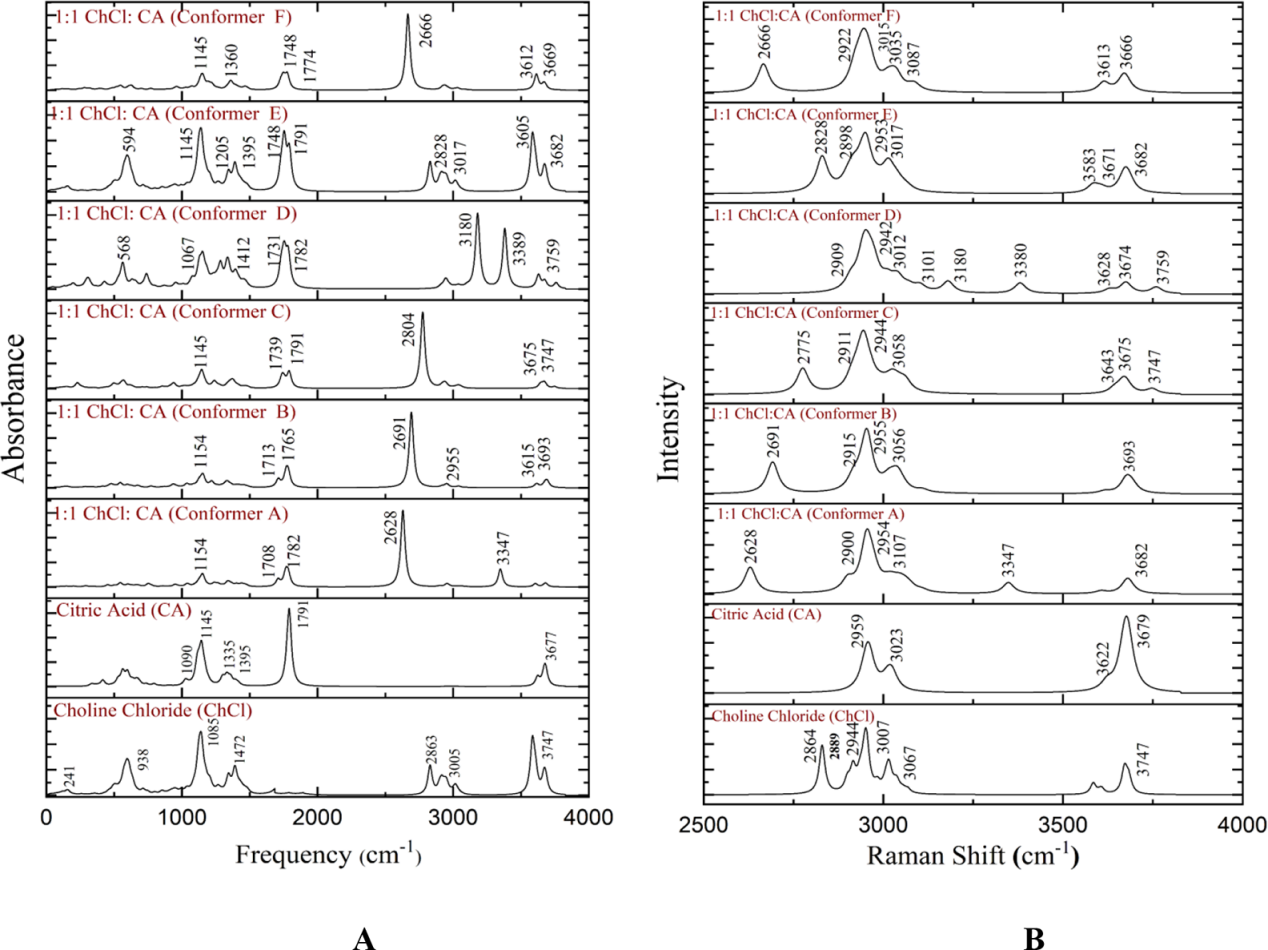
Calculated (A) IR spectra (0–4000 cm^–1^) and (B) Raman spectra (2500–4000 cm^–1^) of six 1:1 ChCl/CA DES conformers.

A noticeable peak observed at 1791 cm^–1^, which matches the C=O stretching, exhibits splitting into three or more in all DES conformers within the range of 1698–1808 cm^–1^. This greatly suggests the formation of DESs (Figure 5A and Table S2). We detected another noticeable change that is the O–H stretching of the carboxylic group of CA ranging from 3622 to 3680 cm^–1^, which has been broadening or nearly merged while forming DESs. Within the frequency band of 1299–1414 cm^–1^, there exists some correspondence to the −CH_2_ and −OH rocking of the carboxylic group, as well as the −CH_2_ bending in CA. This bending is observed to be nearly merged in conformers A, B, and C while producing intense peaks in conformer E and one steep peak in conformer F. These observations provide evidence of the formation of DESs. Observing the IR spectrum of ChCl, −CH_3_ symmetric and asymmetric stretching of the quaternary ammonium group and CH_2_ stretching have been found in the frequency range 2863–3005 cm^–1^, completely disappeared, or merged (conformer E), which is a staunch confirmation of forming DESs.

In the Raman spectra of all six conformers (Figure 5B and Table S3) labeled as A, B, C, D, E, and F, peaks have been broadened or intended to be merged compared to the vibrational features that have appeared in ChCl and CA individually between 2864 and 3067 cm^–1^ and 2959 and 3023 cm^–1^ correspondingly. The peaks detected at 2959–3101 cm^–1^ represent several stretching including CH_3_ symmetrical and asymmetrical stretching of the quaternary ammonium group of ChCl, CH_2_ symmetrical and asymmetrical stretching of ChCl, and CH_2_ asymmetric stretching of CA because of H-bond formation between ChCl and CA. Moreover, one peak generated for C=O str. in the carboxylic group of CA at 1791 cm^–1^ in DESs has appeared with two or three peaks produced for the same corresponding vibration (C=O str.) at a different frequency and has slightly merged or broadened immensely as happened in IR spectra of all DES conformers. The IR and Raman spectra of the 1:1 ChCl/CA cluster conformers were analyzed and contrasted to the spectra of ChCl and CA. The results indicated the hydrogen bond formation, as certain peaks in the conformer’s spectrum were either significantly reduced or merged.

### Conclusions

In conclusion, the implementation of DSC, ATR-FTIR, Raman spectroscopy, and ^1^H NMR has enabled a comprehensive assessment of the formation, interactions, and structural aspects of the constituents in 1:1 ChCl/CA DESs. The visualization of structural orientations and interactions of eutectic systems implementing molecular dynamics and DFT methods is crucial in determining their chemical and physical characteristics, as the nature of DES component bonding and network shows a noteworthy role in these aspects. The findings of combined approaches suggest that the peaks viewed in the DES spectrum are associated with the O–H stretching vibration, whereas Cl^–^ serves as a mediator to link ChCl and CA, thereby forming the DES cluster. The broad spectrum of O–H for DESs demonstrates the formation of cluster conformers by H-bonding. In addition, the C–H bending peak at 1145 cm^–1^ in the fingerprint region, which was unique to the ChCl spectra and absent from the CA spectrum, suggests that ChCl exerts a substantial influence on cluster conformers. In all experimental and theoretical analyses, the Raman spectra indicated comparable outcomes, with vibrational changes in the DES cluster indicating the compact nature of the 1:1 ChCl/CA DES cluster. The strong hydrogen bond interactions reported in conformer E are supported by ^1^H NMR investigations, which reveal that the electronegative atom, chloride (Cl^–^), interacts and forms strong hydrogen bonds with H3 and H4 of the methyls and methylene groups of ChCl, respectively. ^1^H NMR examinations also revealed minor chemical shift variations for H2 of ChCl, which may be explained by the weak interaction of H2 with CA’s hydroxyl oxygen found in cluster E. H1 of ChCl had the greatest chemical shift variation, which explains the strong hydrogen bond of H1 with one of CA’s carbonyl oxygens as seen in cluster E. Overall, the findings of this study offer enhanced comprehension of the interactions and structural features of the components within choline chloride-based DESs, and this will help to optimize and tune DESs for various applications.

## Supporting Information Available

Single point energy (SPE) calculations for all 14 conformers (Table S1); calculated IR wavenumbers of ChCl, CA, and all six conformers (Table S2); calculated Raman shift of ChCl, CA, and all six conformers (Table S3); comparison between YASARA autoutilized and external Autosmiles Gaff2 force field parameters in MD simulation in terms of energetic terms (Figure S1); comparative interaction analysis between initial MD simulations (Figure S2); RDF diagrams of DES systems between different atom pairs (Figure S3); and IR spectrum of ChCl, CA, and five different ratios of ChCl and CA and the different ratio DESs (1:1, 1:2, 1:3, 2:1, and 3:1 ChCl/CA) (Figure S4) (PDF)
