# Supplementary material for: Elucidating the Structure, Dynamics, and Interaction of a Choline Chloride and Citric Acid Based Eutectic System by Spectroscopic and Molecular Modeling Investigations
Source: ACS Omega. 2023 Oct 4;8(41):38243–51. doi: 10.1021/acsomega.3c04570 (PMC10586180; doi:10.1021/acsomega.3c04570)
Supplement: Supplementary file 1 — ao3c04570_si_001.pdf [file ao3c04570_si_001.pdf]

Elucidating Structure, Dynamics, and Interaction of Choline Chloride and Citric Acid Based Eutectic System by Spectroscopic and Molecular Modelling Investigations

Md Ackas Ali[a], Md Abdul Kaium[b], Sayed Nesar Uddin[b], Md Jaish Uddin[b], Oluseyi Olawuyi[a], Albert D. Campbell Jr.[a], Carl Jacky Saint-Louis[a], Mohammad A. Halim[a]*

[a]Department of Chemistry and Biochemistry, Kennesaw State University, Kennesaw, GA, 30144 USA

[b]Division of Quantum Chemistry, The Red-Green Research Center, BICCB, 16, Tejkunipara, Tejgaon, Dhaka 1215, Bangladesh

*Corresponding Author: Mohammad A. Halim, Email: mhalim1@kennesaw.edu; Phone: Phone: 470-578-6759.

Supporting Information (SI)

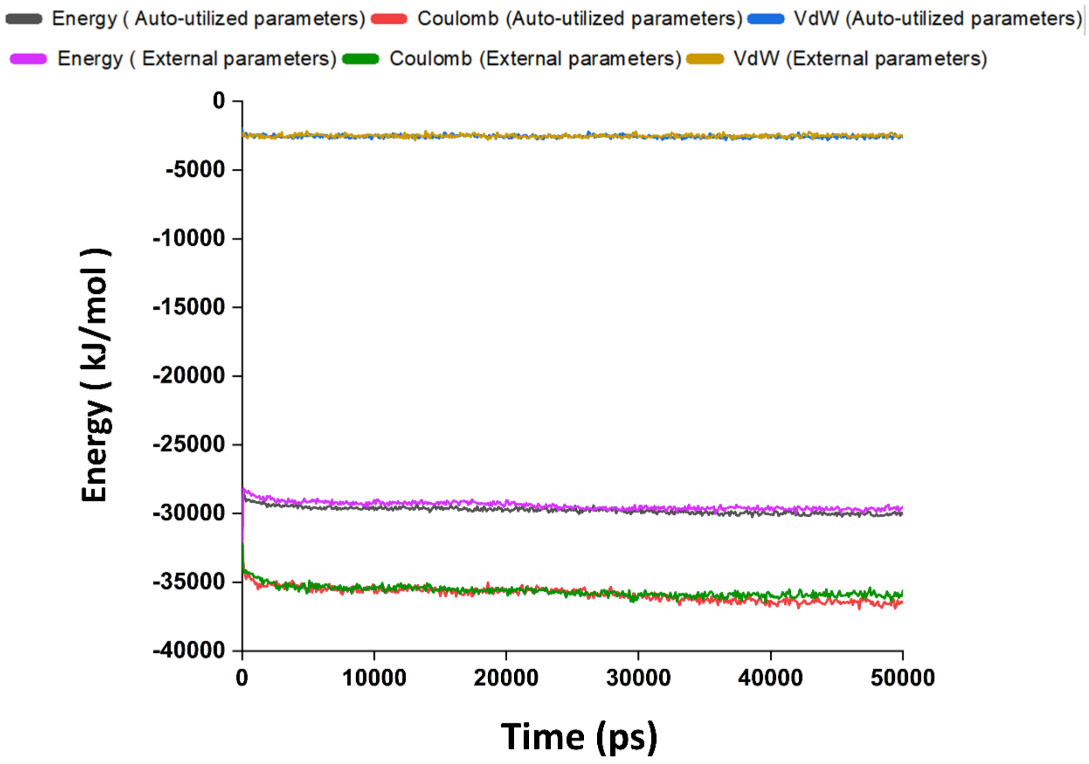

Figure S1: Comparison between YASARA auto-utilized and external Autosmiles Gaff2 forcefield parameters in MD simulation in terms of energetic terms.

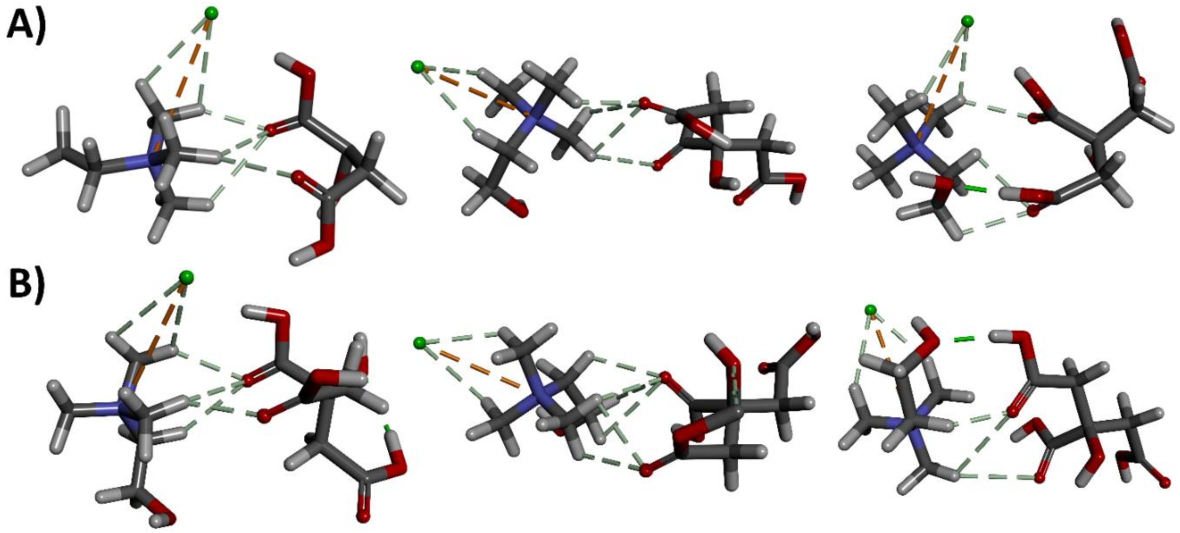

Figure S2: Comparative interactions analysis between (A)YASARA auto-utilized and (B) external Autosmiles Gaff2 forcefield parameters in MD simulation.

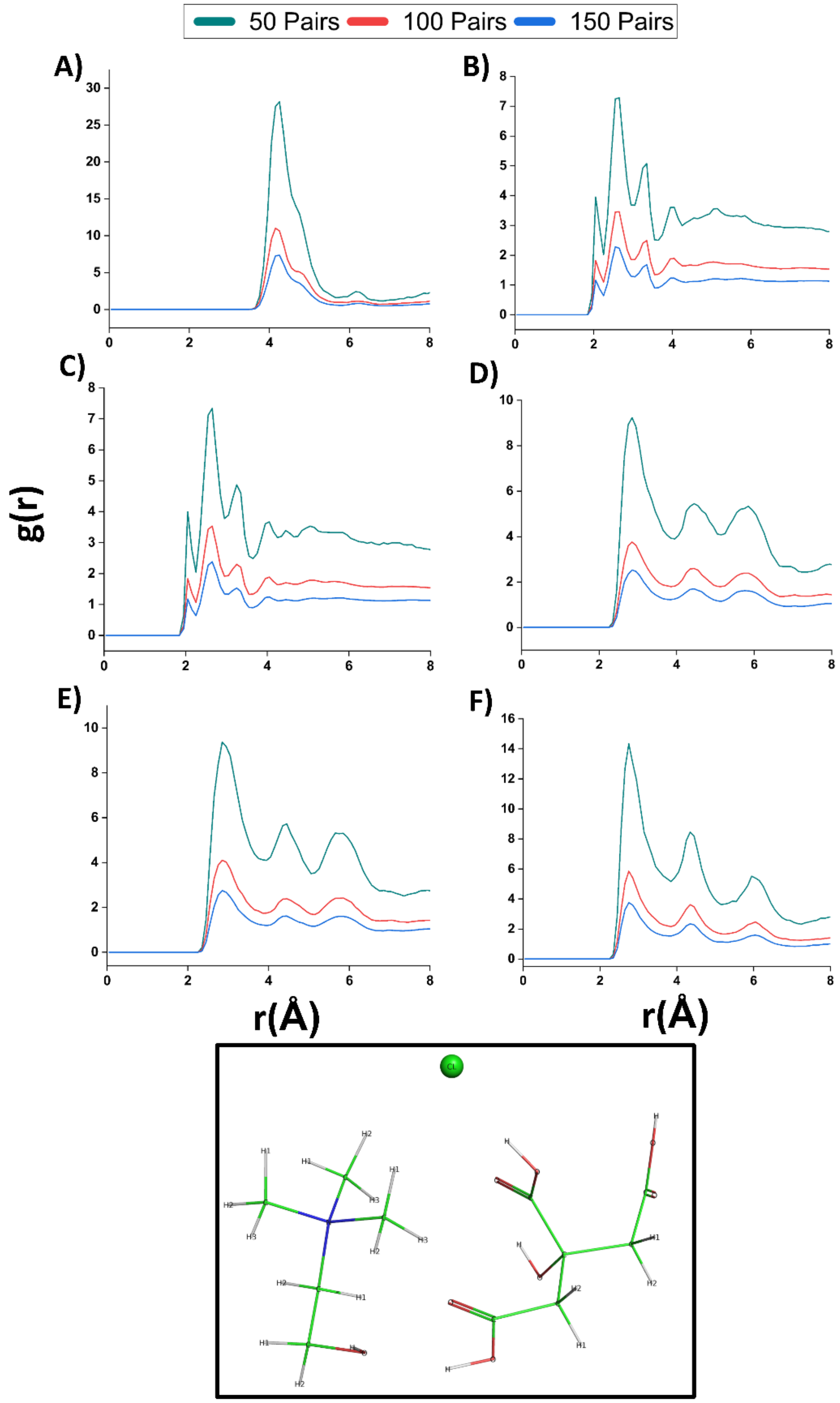

Figure S3: RDF diagrams of des systems between different atom pairs. (A) N [CH] – CL, (B) H1 [CH] – O [CA], (C) H2 [CH] -O [CA], (D) H1 [CH] -CL, (E) H2 [CH] -CL and (F) H3 [CH] – CL. {CA* Citric Acid , CH* Choline }

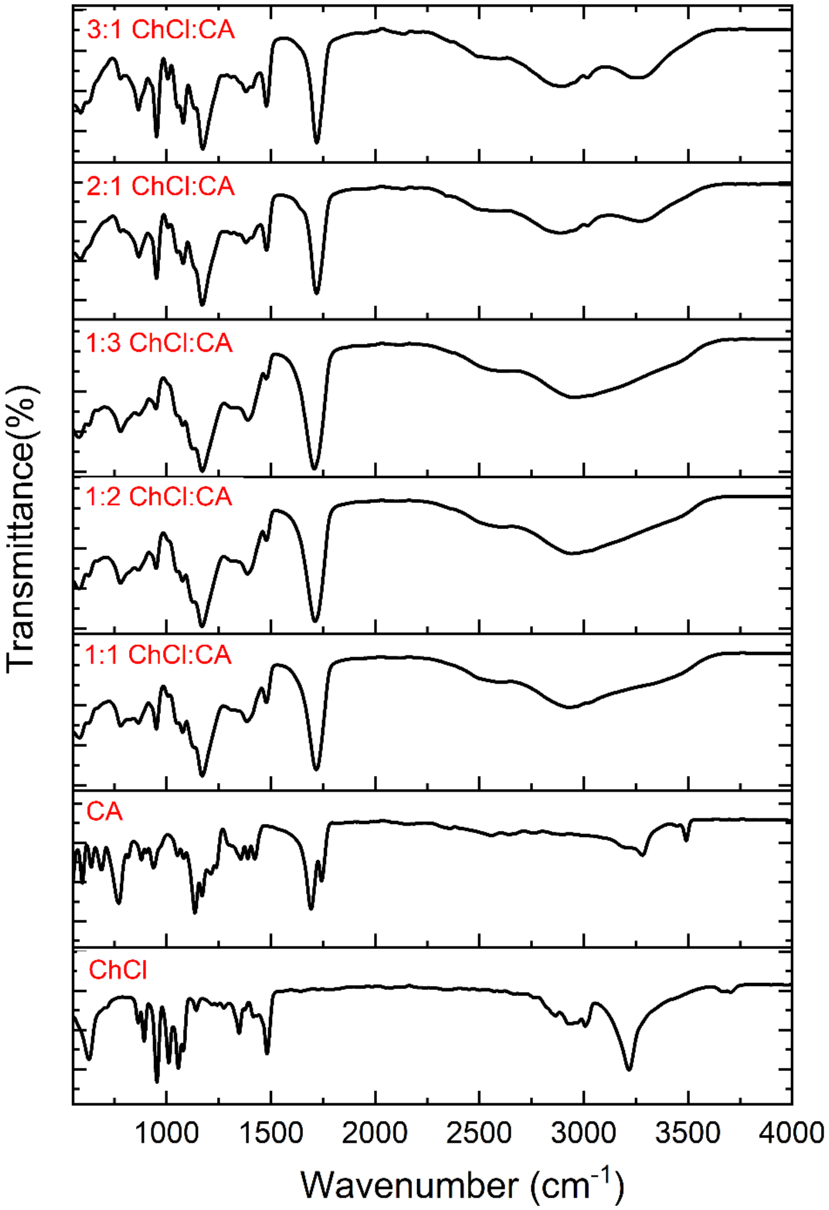

Figure S4: The IR Spectrum of ChCl, CA, and 5 different ratios of ChCl and CA and the different ratio DESs (1:1 ChCl: CA, 1:2 ChCl: CA, 1:3 ChCl: CA, 2:1 ChCl: CA, 3:1 ChCl: CA).

List of Supporting Tables

Table S1: The Single Point Energy (SPE) calculations for all 14 conformers to identify the best possible conformers for further study. All 6 conformers with negative energy were subsequently considered for quantum calculations in the DFT-wB97XD/6-311G++ (d,p) level of theory. (H = Hartree unit)

Name	Energy(H) SPE	Energy(H) SPE (CA)	Energy(H) SPE (ChCl)	Energy(H) diff.	Energy(kj) diff.
50 pairs00500.1	-1542.192518	-755.944789	-786.240093	-0.007636	-20.04831953
50 pairs00500.1.1	-1542.169942	-755.940858	-786.217366	-0.011718	-30.76561134
50pairs00500.1.2	-1542.146907	-755.945137	-786.209872	0.008102	21.2718026
				0	
50 pairs00500.2	-1542.201541	-755.951944	-786.238656	-0.010941	-28.72559769
50 pairs00500.2.1	-1542.17619	-755.95167	-786.185456	-0.039064	-102.5625398
				0	
50 pairs00500.3	-1542.18913	-755.945393	-786.245487	0.00175	4.5946254
50 pairs00500.3.1	-1542.196081	-755.951975	-786.230171	-0.013935	-36.58634529
50 pairs00500.3.2	-1542.139743	-755.945157	-786.199121	0.004535	11.9066434
				0	
50 pairs00500.4	-1542.192454	-755.952311	-786.240046	-0.000097	-0.254673519
50 pairs00500.5	-1542.18581	-755.947186	-786.240995	0.002371	6.22506097
50 pairs00500.5.1	-1542.192753	-755.951582	-786.23641	-0.004761	-12.50000645
				0	
50 pairs00500.6	-1542.171	-755.945252	-786.229689	0.003941	10.3470963
50 pairs00500.6.1	-1542.179087	-755.945399	-786.239981	0.006293	16.5222728
50 pairs00500.6.2	-1542.170425	-755.940683	-786.230766	0.001024	2.6885122

Table S2. IR in frequencies of choline chloride (ChCl), citric acid (CA), and Six cluster conformers (A, B, C, D, E, and F) of 1:1 ChCl: CA in DESs system in the DFT-wB97XD/6-311G++ (d,p) level of theory.

**ChCl**		**CA**		**Conformer A**		**Conformer B**		**Conformer C**		**Conformer D**	
**A[bonds]**	**Fre (cm^{-1})**	**A[bonds]**	**Fre (cm^{-1})**	**A[bonds]**	**Fre (cm^{-}**	**A[bonds]**	**Fre (cm^{-1})**	**A[bonds]**	**Fre (cm^{-1})**	**A[bonds]**	**Fre (cm^{-1})**
O(7)-H(22) bending	215	C(10)-H_2 twst. /O(1)-H(18) roc ./O(2)-H(19) roc ./ O(5)-H(21) roc.	1112	C(33)-O(25) str. / O(24)-H(41)	1708	C(33)-O(25) str. / O(24)-H(41) (CA)	1710	C(33)-O(25) str. / O(24)-H(41) (CA)	1790	C(33)-O(25) str. / O(24)-H(41) (CA)	1754
CH_3 symm. str.	2863	C(13)=O(7) str. / O(5)-H(21)	1789	C(34)=O(28) s roc. (CA)	1764	C(34)=O(28) str. / O(26)-H(42) roc. (CA)	1767	C(34)=O(28) str. / O(26)-H(42) roc. (CA)	1753	C(34)=O(28) str. / O(26)-H(42) roc. (CA)	1783
CH_2 asy. Str.	3007	C(12)=O(6) str. / O(4)-H(20) roc.	1775	C(35)=O(29) s roc. (CA)	1782	C(35)=O(29) str. / O(27)-H(43) roc. (CA)	1863	C(35)=O(29) str. / O(27)-H(43) roc. (CA)	1737	C(35)=O(29) str. / O(27)-H(43) roc. (CA)	1733
O(7)-H(22) str.	3747			O(24)-H(41) str. (CA)	2628	O(24)-H(41) str. (CA)	1782	O(27)-H(43) str. (CA)	2775	O(27)-H(43) str. (CA)	3180
				O(7)-H(22) str. (ChCl)	3347	O(7)-H(22) str. (ChCl)	3693	O(26)-H(42) str. (CA)	3667	O(24)-H(41) str. (CA)	3380
				O(23)-H(40) str.(CA)	3605	O(23)-H(40) str. (CA)	3615	O(23)-H(40) (CA)	3643	O(23)-H(40) (CA)	3628
				O(27)-H(43) $_{COOH}$ str. (CA	3682			O(24)-H(41) str. (CA)		O(26)-H(42) (CA)	3694
										O(7)-H(21) str. (CA)	3759

**Conformer E**		**Conformer F**	
**A[bonds]**	**Fre (cm^{-1})**	**A[bonds]**	**Fre (cm^{-1})**
C(33)-O(25) str. / O(24)-H(41) roc. (CA)	1791	C(33)-O(25) str. / O(24)-H(41) roc./ C(35)=O(29) str. / O(27)-H(43) roc. (CA)	1729-1747
C(34)=O(28) str. / O(26)-H(42) roc. (CA)	1732	C(34)=O(28) str / O(26)-H(42) roc. (CA)	1776
C(35)=O(29) str. / O(27)-H(43) roc. (CA)	1754	O(24)-H(41) str.	2665
C(31)-H(37) str. (CA)	2828	O(8)-H(22) str. (ChCl)	3612
CH_3 symm. str. (ChCl)	2898		
CH_2 symm. str. (ChCl)	2934		
O-H_{COOH} roc ./CH_2 tws. (CA)	1070		
CH_2 asymm. str. (ChCl)	3017		
O(7)-H(22) str. (ChCl)	3584		
O(23)-H(40) str. (CA)	3606		
O(27)-H(43) str.(CA)	3671		
O(24)-H(41) str.(CA)	3682		

Table S3: Raman shifts of ChCl, CA, and Six cluster conformers (A, B, C, D, E, and F) of 1:1 ChCl: CA in DESs in the DFT-wB97XD/6-311G++ (d,p) level of theory.

**ChCl**		**CA**		**Conformer A**		**Conformer B**		**Conformer C**		**Conformer D**	
**A**^{bonds}	**Fre (cm^{-1})**	**A**^{bonds}	**Fre (cm^{-1})**	**A**^{bonds}	**Fre (cm^{-1})**	**A**^{bonds}	**Fre (cm^{-1})**	**A**^{bonds}	**Fre (cm^{-1})**	**A**^{bonds}	**Fre (cm^{-1})**
O(7)-H(22) str.	3747	C(11)=O /C(12)=Ostr. O(2)-H roc. O(4)-H roc.(COOH)	1791	O(24)-H(41) str. (CA)	2628	O(24)-H(41)	2691	O(27)-H(43) str. (CA)	2775	C(3)-H_2 sym. str. (ChCl)	2909
CH_3 symm. str.	2898	C(10)-H_2 sym./ C(9)-H_2 sym. Str.	2959	C(3)-H_2 sym. Str.	2899	C(3)-H_2 str.	2915	C(1)-H_2 str. C(6)-H_3 str. (ChCl)	2944	C(5)-H_3/ C(1) -H_2 sym. Str. (ChCl)	2942
C(3)-H_2 asy. Str.	2910	C(9)-H_2 asym. Str	3009	C(1)-H_2 str. / C(6)-H_3 str. /C(4)-H_3 str ./C(3)-H_2 asy. Str. (CA)	2954	C(5)-H_3 sym. Str./C(1)-H_2 str. (ChCl)	2955	C(32)-H_2 asy. Str. (CA)	3025	C(5)-H_3 sym. Str. (ChCl)	2951
C(1)-H_2 sym. Str.	2944	C(10)-H_2 asym. Str	3023	C(5)-H_3 sym. Str. (ChCl)	2971	C(32)-H_2 asy. Str.	3017	C(4)-H /C(6)-H asy. Str.	3058	C(4)-H_3 sym. Str. (ChCl)	2974
C(4)-H_3/ C(4)-H_2	3005	O(4)-H(20)$_{COOH}$	3669	C(32)-H_2 asym. Str.	3018	C(4)-H_2/C(5) -H_2 asy. Str.	3038	O(23)-H(40) $_{COOH}$ str. (CA)	3643	O(27)-H(43) str . (CA)	3180
		O(2)-H(19)$_{COOH}$/ O(5)-H(21)$_{COOH}$	3680	O(7)-H(22) str. (CA)	3347	C(6)-H_2/ C(4)-H_2 asy.	3043	O(26)-H(42) $_{COOH}$ str. (CA)	3667	O(24)-H(41) str. (CA)	3380
				O(27)-H(43) str. (CA)	3682	O(26)-H(42)$_{COOH}$ str. (CA)	3673	O(24)-H(41) $_{COOH}$ str. (CA)	3675	O(23)-H(40) str. (CA)	3628
				O(26)-H(41) str. (CA)	3676	O(27)-H(43) str. (CA)	3681	O(7)-H(22) str. (CA)	3747	O(26)-H(42) str. (CA)	3674
										O(7)-H(21) str. (ChCl)	3759

**Conformer E**		**Conformer F**	
**A**^{bonds}	**Fre** (cm^{-1})		
C(31)-H_2 sym. Str.	2828	O(24)-H(41) str. (CA)	2665
C(1)-H_2 str. (ChCl)	2953	C(5)-H_3 sym. Str./ C(6)-H_3 sym. Str.	2950
C(5)-H_3 str. (ChCl)	2915	O(8)-H(22) str. (ChCl)	3612
O(7)-H(22) str. (ChCl)	3584	O(26)-H(42) str. (CA)	3672
O(23)-H(40) Str. (CA)	3606	C(7)-H_3 sym. Str.	2922
O(26)-H(42) str. (CA)	3670	C(4)-H_2 asy. Str.	2963
O(24)-H(41) str. (CA)	3682		
